# Norovirus Narita 104 Virus-Like Particles Expressed in *Nicotiana benthamiana* Induce Serum and Mucosal Immune Responses

**DOI:** 10.1155/2014/807539

**Published:** 2014-05-11

**Authors:** Lolita George Mathew, Melissa M. Herbst-Kralovetz, Hugh S. Mason

**Affiliations:** ^1^Center for Infectious Diseases and Vaccinology (CIDV), The Biodesign Institute at Arizona State University, 1001 South McAllister Avenue, Tempe, AZ 85287, USA; ^2^The School of Life Sciences, 1001 South McAllister Avenue, Tempe, AZ 85287, USA; ^3^Department of Basic Medical Sciences, University of Arizona College of Medicine-Phoenix, 425 N. 5th Street, Phoenix, AZ 85004, USA

## Abstract

Narita 104 virus is a human pathogen belonging to the norovirus (family Caliciviridae) genogroup II. Noroviruses cause epidemic gastroenteritis worldwide. To explore the potential of developing a plant-based vaccine, a plant optimized gene encoding Narita 104 virus capsid protein (NaVCP) was expressed transiently in *Nicotiana benthamiana* using a tobacco mosaic virus expression system. NaVCP accumulated up to approximately 0.3 mg/g fresh weight of leaf at 4 days postinfection. Initiation of hypersensitive response-like symptoms followed by tissue necrosis necessitated a brief infection time and was a significant factor limiting expression. Transmission electron microscopy of plant-derived NaVCP confirmed the presence of fully assembled virus-like particles (VLPs). In this study, an optimized method to express and partially purify NaVCP is described. Further, partially purified NaVCP was used to immunize mice by intranasal delivery and generated significant mucosal and serum antibody responses. Thus, plant-derived Narita 104 VLPs have potential for use as a candidate subunit vaccine or as a component of a multivalent subunit vaccine, along with other genotype-specific plant-derived VLPs.

## 1. Introduction

Noroviruses (NoVs), which belong to the family Caliciviridae, are the leading causes of acute viral gastroenteritis in humans worldwide. NoVs are approximately 38 nm icosahedral viruses and contain a positive-sense, single-stranded, nonenveloped RNA genome of ~7.5 kb [1]. The open reading frame (ORF) 1 encodes nonstructural proteins required for viral replication. ORF2 codes for the capsid protein and the ORF3 encodes a small basic protein that is a minor structural protein included in the virion [2–4]. The major capsid protein can diverge by as much as 60% between genogroups and 20–30% between genotypes within a genogroup. Human NoVs are classified into two distinct genogroups (GI and GII) [5] which are further subdivided into more than 25 different genotypes. The majority of norovirus outbreaks currently are caused by the GII.4 genotypes, which cause significant morbidity and mortality [6]. Several viruses in this category have emerged since 1990s worldwide [7–15]. Thus, we studied the development of a plant-based vaccine against Narita 104 virus (NaV) (NCBI database, accession number AB078336), a GII.4 strain of norovirus [16]. 

Heterologous expression of recombinant Norwalk virus capsid protein (NVCP) in insect and plant systems showed that the capsid protein can self-assemble into virus-like particles (VLP) that are morphologically and antigenically similar to native NV particles [17–19]. Recombinant Norwalk virus VLP (rNV VLP) elicited VLP-specific systemic (serum IgG) and mucosal (vaginal and fecal IgA) antibodies in mice when administered orally in presence and in the absence of an adjuvant [18, 20, 21]. The plant-based expression of native NaV capsid protein was poor due to incorrect mRNA processing, but a plant optimized gene enhanced expression [22]. Also, new approaches to achieving high levels of protein using rapid virus-vectored transient expression have been developed, including the tobacco mosaic virus (TMV) system (ICON system) [23–25].

A commercial vaccine to prevent norovirus infection is not currently available. For the successful design of a norovirus vaccine, variability of capsid protein in different genotypes must be taken into account [16, 17, 26–28]. Plants are an economical and safe platform for vaccine development due to ease of scalability and lack of mammalian pathogens [29, 30]. The ICON system allows for rapid and scalable production of various antigens. Thus, within a short period of time multiple vaccine candidates can be expressed and tested for their efficacy [21, 31–34].

In this study, we performed plant-based expression and partial purification of Narita VLP (NaVLP) and further evaluated these VLPs as a candidate vaccine by testing the immunogenicity and induction of GII.4-specific VLP antibody in mice.

## 2. Materials and Methods

### 2.1. Construction of Plant Expression Vector

A plant-optimized sNaVCP gene (Genbank accession number GQ389627) from pCRblunt-sNaVCP [22] was introduced into pICH10990 (ICON Genetics, Halle, Germany) to obtain pICHsNaV. The coding sequence in pCRblunt-sNaVCP was end-tailored to create an EcoRI site at the 5′ end using a high-fidelity PCR kit (Roche) with primers sNaCP-eco (5′-GACGAATTCAACAATGAAGATGGCTTCTAATG) and M13RHT (5′-GGAAACAGCTATGACCATG). The resulting PCR product was digested with EcoRI-SacI and the fragment was ligated into pICH10990 digested likewise to yield pICHsNaV (3′ module). The plasmid was sequenced to assure fidelity and mobilized into* Agrobacterium tumefaciens* GV3101. The two modules containing the integrase (pICH14011) and the 5′ module (pICH15879) that mediates cytosolic accumulation were coinfiltrated along with the 3′ module [24]. For expression of NVCP and GFP, the 3′ module vectors pICH-sNVCP and pICH-GFP [21] were used.

### 2.2. Agrobacterium Delivery Using Vacuum Infiltration

Equal volumes of overnight-grown* Agrobacterium* cultures (3′ module, integrase, and 5′ module), OD_600_ ≈ 1.8, were mixed and sedimented at 6,000 ×*g* for 3 min. The pellet was resuspended in 15 mL of a solution containing 10 mM MES (pH 5.5) and 10 mM MgSO_4_ in order to obtain the final combined OD600 of ~0.1. Four-week-old greenhouse grown* N. benthamiana *plants were submerged upside down in polystyrene cylindrical flasks (6 cm height, 7 cm diameter) containing 2.5 L of the bacterial suspension and transferred to a desiccator. Vacuum (23 in. Hg) was applied for 2 min using a pump (Welch model number 2546B-01) and gently released. Gentle shaking of the desiccator was performed during vacuum application to remove air bubbles. After confirming the success of the infiltration by visual inspection (infiltrated areas of the leaf become translucent when observed against a source of light), plants were placed in the growth room to recover.

### 2.3. RNA Extraction and Hybridization

Total RNA was extracted from leaves of tobacco (Trizol reagent, Invitrogen) and residual DNA removed by DNA-free system (Ambion). RNA was fractionated on formaldehyde agarose gels and blotted to nylon membranes as described [35]. The membrane was hybridized with digoxigenin-labeled probe synthesized by PCR using primers 3′UTR-F (GAGCTCCCGGGGATCCTCTA) and 3′UTR-R (TTAGGGAGGATTCGAACCTCTCAC) and pICHsNaV as template, as described in the product literature (Roche).

### 2.4. Protein Extraction

Four days postinfiltration (DPI),* N. benthamiana *leaves were harvested, weighed, flash-frozen in liquid N_2_, and stored at −80°C. For total protein extraction, frozen leaves were homogenized by blending in extraction buffer [25 mM sodium phosphate (pH 6.6), 100 mM NaCl, 1 mM EDTA, 50 mM sodium ascorbate, and 10 *μ*g/mL leupeptin] at the ratio of 10 mL per gram of fresh weight of leaf. Homogenates were then immediately filtered through Miracloth (Calbiochem) and centrifuged at 11,300 g for 20 min at 4°C. The supernatant was passed through 0.22 *μ*m filter (Millipore) and kept at 4°C before concentrating using stirred cell (Amicon, model number 8200, Millipore) with 300 kDa cut-off membrane (Ultracel PL30000; Millipore) followed by further filtration through a 30 kDa cut-off membrane (Biomax PB 300,000; Millipore). The retained concentrated extract was used for further analysis.

### 2.5. SDS-PAGE and Western Blot

Samples were denatured by boiling in SDS-PAGE buffer and resolved using 4–20% gradient polyacrylamide gels and followed by either Coomassie brilliant blue G-250 staining with PageBlue protein staining solution (Fermentas) or electrophoretically transferred to Hybond P membrane (Amersham Pharmacia) for Western blot. Membranes were probed with rabbit polyclonal anti-NaV antibody conjugated to HRP diluted 1 : 5000. The membranes were developed by chemiluminescence using the ECL plus detection reagent (Amersham Pharmacia).

### 2.6. NaVCP ELISA and Bradford

Plant extracts were analyzed by NaVCP sandwich ELISA. Recombinant NaVCP derived from insect cells using baculovirus system (i-NaV, gift of N. Takeda) served as reference standard. Briefly, rabbit anti-NaV 104 serum was diluted to 1 : 10,000 in 0.01 M phosphate-buffered saline (PBS; 50 *μ*L per well) and coated on the 96-well plate using carbonate high binding buffer for 4 h at 23°C. The plates were then blocked with 5% nonfat dry milk in PBS with 0.05% Tween 20 (DM/PBST) for 1 h at 23°C. After washing the wells once with 1X PBST, samples (50 *μ*L per well) diluted in 1%DM/1X PBST were added and incubated for 1 h at 37°C. The wells were washed three times and incubated for 2 h at 37°C with rabbit anti-NaV104 IgG-horseradish peroxidase conjugate, diluted 1 : 20,000 in 1%DM/PBST. The plate was developed with TMB detection kit (Amersham Biosciences) for 8–10 min at 23°C and the reaction was ended by adding 1 M H_3_PO_4_. Absorbance was read at 450 nm. For standard curve, i-NaV was diluted serially twofold in 1%DM/1X PBST from concentrations starting at 12.5 ng/mL and processed as above. The leaf supernatants were tested for total soluble protein (TSP) by the Coomassie blue dye-binding assay [36] using reagent (Bio-Rad) with BSA as a standard.

### 2.7. Sucrose Gradient Sedimentation

The sucrose gradient was performed as described [21] with modifications. Briefly, gradients were generated using 60, 50, 40, 30, 20, and 10% sucrose prepared in 0.01 M PBS and incubated for at least 2 h at 4°C to allow for formation of continuous gradient. The sample (either crude leaf extract or previously purified NaVLP) was layered on the top of the gradient, and tubes were centrifuged at 151,000 g for 2.5 h at 4°C. Fifteen fractions were generated and analyzed by ELISA.

### 2.8. Electron Microscopy

Pooled sucrose gradient fractions were analyzed by antibody immobilization negative staining and examination by transmission electron microscopy (TEM). Formvar coated Ni grids were coated with rabbit polyclonal anti-NaV antibody (1 : 10 dilution) for 10 min at 23°C. Samples were applied to antibody-coated grids for 10 min. After washing, grids were stained using 2% uranyl acetate for 1 min and examined with a Philips CM-12 TEM.

### 2.9. Anion Exchange Chromatography

Anion exchange chromatography was performed as described [37] with slight modifications. The concentrated sample was applied to packed columns (1.5 × 12 cm, Bio-Rad) containing DEAE Sepharose Fast flow (GE Healthcare) that was preequilibrated with buffer (25 mM sodium phosphate, 100 mM NaCl, pH 5.8). The flow-through containing NaVCP products were collected separately after washing five times with the same buffer. Final elution was done with high salt buffer (2 M NaCl in 25 mM sodium phosphate buffer, pH 5.8) and collected separately. The flow-through fractions containing most of the NaVCP products were pooled and then concentrated again using 30 kDa cut-off membrane (Centriprep YM-30, Millipore) and analyzed by SDS-PAGE gel and ELISA. The partially purified sample was used for immunization studies.

### 2.10. NaVCP Immunization in Mice

All animals were housed in American Association for Laboratory Animal Care, approved quarters, and were provided unlimited access to food and water. All procedures were approved by the ASU IACUC and performed in accordance with the Animal Welfare Act. Female 5-week-old BALB/c mice (Charles River, San Diego, CA) were distributed randomly and acclimated for at least 1 week prior to any procedures or treatment. Mice (*N* = 10/group) were immunized intranasally with NaVCP VLPs (25 *μ*g) alone on days 0 and 21 and compared to mock-vaccinated (PBS alone) controls. Mice were not anesthetized for nasal immunization. Intranasal immunization was performed by using a 20 *μ*L pipet to instill half of the vaccine into each nare (~5–10 *μ*L/nare). Serum was collected during the entire timecourse (days 0, 12, 21, 42, and 56) as previously described [38]. After the animals were humanely euthanized, nasal lavages were collected and processed as previously described [38]. All samples were stored at −80°C for future analysis of antibody titers. Serum and nasal samples were evaluated by ELISA as previously described [38] and outlined above. Sample dilutions included the following ranges: serum (1 : 100–1 : 10,000,000) and nasal samples (1 : 2–1 : 5000). An absorbance value of 0.1 or higher was considered to be positive and the geometric mean titer (GMT) was calculated as the reciprocal of the highest dilution tested that provided a positive absorbance value.

### 2.11. Statistical Analyses

Statistical analysis was performed using Prism software (GraphPad; San Diego, CA). Geometric mean titer (GMT) values were evaluated statistically at each time point using the Mann-Whitney nonparametric test. Statistical comparisons between the NaVLP vaccination group and the PBS delivery (mock-vaccinated) group were completed and displayed in Figure 4. A* P* value < 0.05 was considered statistically significant.

## 3. Results

### 3.1. Transient Expression of NaVCP in* Nicotiana benthamiana*

**Figure 1:**
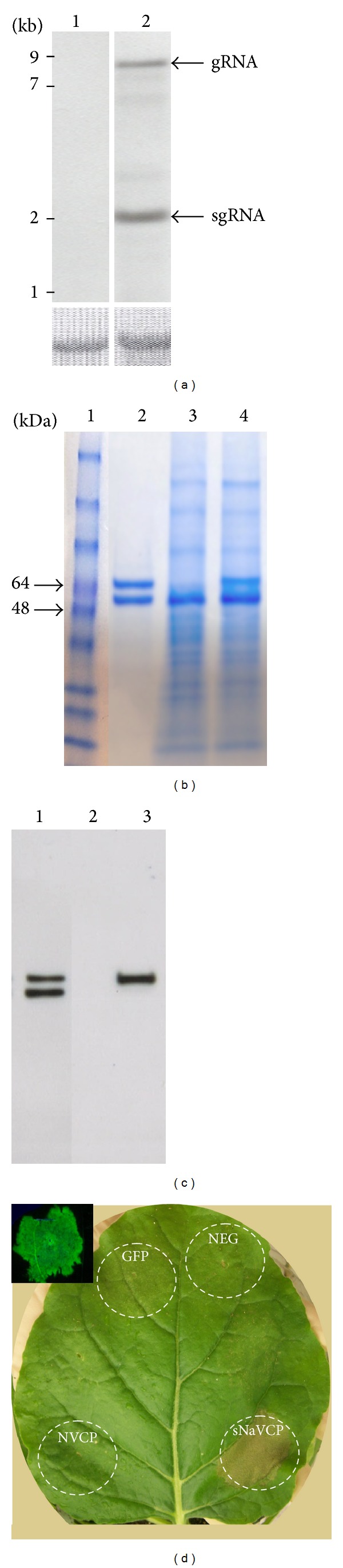
Initial characterization of transient expression of NaVCP. (a) Northern blots of RNA from leaves infiltrated with pICHsNaV (lane 2) showing genomic RNA (gRNA) and subgenomic RNA (sgRNA) of NaVCP after 3 DPI. Lane 1 is noninfiltrated leaf as negative control. Two *μ*g of total RNA was loaded and probed with probe specific to TMV 3′UTR. Ribosomal RNA loading is shown at the bottom. (b) Coomassie stained SDS-PAGE of NaVCP showing presence of NaVCP at expected size ~58 kDa. Lane 1: protein molecular mass markers; lane 2: insect-derived NaVCP (1 *μ*g); lane 3: crude protein extract (15 *μ*g) from leaves infected with empty vector; lane 4: crude protein extract (15 *μ*g) from leaf samples infiltrated with pICHsNaV and harvested at 4 DPI. The 64 kDa and 48 kDa molecular mass markers are indicated by arrows at left. (c) Western blot of SDS-Page of sNaVCP showing presence of NaVCP at expected size ~58 kDa. Lane 1: insect-derived NaVCP (25 ng); lane 2: crude protein extract (10 *μ*g) from leaves infected with empty vector. Lane 3: crude protein extract (10 *μ*g) from leaf samples infiltrated with pICHsNaV and harvested at 4 DPI. (d) Hypersensitive response on pICHsNaV infiltrated region. Part of the leaf that was infiltrated with pICHsNaV displayed cell death 5 DPI whereas such symptoms are absent in infiltrated areas of GFP, NVCP, and empty vector (Neg). Inset shows GFP fluorescence at 10 DPI.

A TMV-based ICON expression vector was utilized for efficient expression of NaVCP. This TMV vector is based on a deconstructed viral system for expression of gene of interest [21, 24, 25, 34]. The NaVCP coding sequence was cloned into the 3′ module, and when delivered in combination with 5′ module and integrase module, the system drives the expression of NaVCP in the cytoplasm. Three days postinfiltration (DPI), total RNA was extracted from the* N. benthamiana* leaf and RNA blot was performed with 3′UTR specific probe. Both NaVCP transcripts (genomic RNA, ~8 kb and subgenomic RNA, ~2 kb) at expected sizes (Figure 1(a)) were detected. The same RNA blot was restriped and reprobed with NaVCP specific probe (data not shown) to confirm the results. Although high expression of foreign proteins has been obtained using the ICON system, NaVCP expression in plants promoted the rapid onset of tissue necrosis at ~5–6 DPI (Figure 1(d)). In spite of the need to harvest at 4 DPI (much earlier than NVCP, [21]), approximately 0.28 mg of NaVCP per g fresh leaf weight was obtained, as quantified by NaVCP ELISA (Table 1). Plant-derived NaVCP was also detected in the Coomassie stained SDS-PAGE gels loaded with crude protein extracts from* N. benthamiana* (Figure 1(b), lane 4) and was confirmed by Western blot probed with polyclonal rabbit anti-NaVCP antibody (Figure 1(c), lane 3). The plant-derived NaVCP comigrated with insect cell-derived NaVCP at the expected size of 58 kDa (Figure 1(b) lane 2 and Figure 1(c) lane 1) and was absent in leaves infiltrated with an empty vector (Figure 1(b) lane 3 and Figure 1(c) lane 2). The insect-derived VLP sample also had a smaller ~50 kDa protein, which is consistent with insect-based expression of various norovirus capsids and attributed to either an alternative translation initiation site or to proteolytic cleavage [39]. Thus, the plant-based system provided an advantage in that only full-length NaVCP was observed.

### 3.2. Plant-Derived NaVCP Assembles VLPs

**Figure 2:**
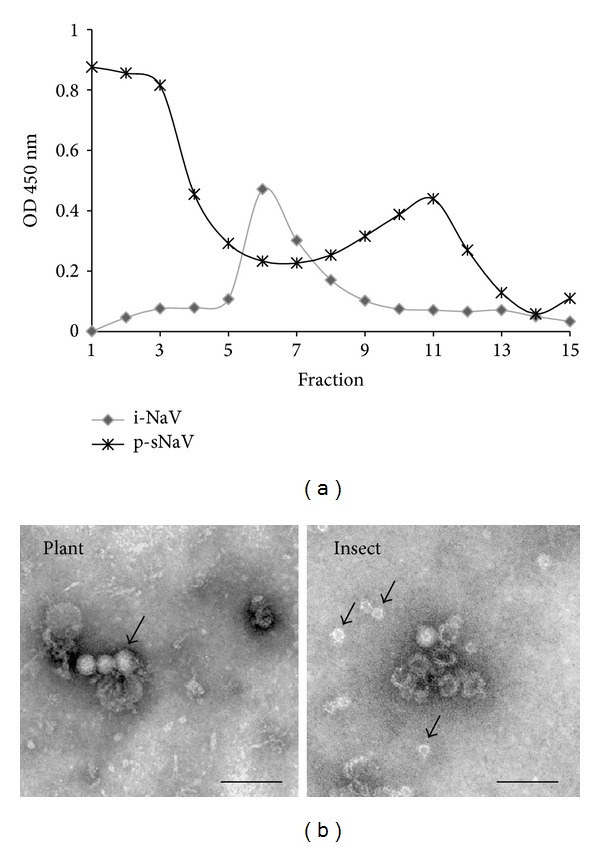
NaVLP assembly and electron microscopy. (a) Sucrose gradient sedimentation for evaluation of VLP assembly for plant- and insect-derived NaVLP. Crude extracts from* N. benthamiana* infiltrated with pICHsNaV or purified insect-derived NaVLP were sedimented separately on 10–60% sucrose gradients and 15 fractions were collected. Distribution of NaVCP across the gradient fractions was determined by ELISA. The top of the gradient is at left. Fractions 5 to 14 were pooled for EM studies. (b) Visualization by electron microscopy of NaVLP. Left: plant-derived NaVLP partially purified by sucrose gradient, immunocaptured on grids, and negatively stained; arrow indicates 33 nm VLP. Right: insect-derived NaVLP; arrows indicate small ~20 nm VLP. Bars = 100 nm.

Sucrose gradient sedimentation was performed to confirm VLP assembly of plant-derived NaVCP. Crude protein extracts from* N. benthamiana* and insect derived NaVCP were separately layered on 10–60% sucrose gradients. The gradient fractions (1–15) from top to bottom were analyzed by NaVCP ELISA and also confirmed by Western blot on selected fractions (data not shown). The profiles revealed two peak fractions (~2–3 and ~10–12) for plant-derived NaVCP (Figure 2(a)). Interestingly, plant-derived NaVCP showed a faster sedimenting peak at fraction 11 whereas insect-derived NaVCP exhibited a peak at fraction 6. To examine VLP, pooled faster sedimenting fractions (5–14) from plant samples were immobilized using rabbit anti-NaVCP antibody followed by negative staining and visualized by electron microscopy. Both plant and insect-derived samples showed particles that were approximately 33 nm in diameter (Figure 2(b)) and appeared similar to Norwalk VLP [19, 21, 40]. Both samples but especially the insect-derived sample also contained smaller VLP with a diameter of ~20 nm, consistent with the 23 nm Norwalk VLP obtained from insect cells [19]. The slower sedimenting fractions in the plant samples most likely represent VLP assembly intermediates.

### 3.3. Partial Purification of NaVCP from* N. benthamiana*

In order to conduct immunization studies, various methodologies for purifying plant-derived NaVLP were investigated. In a previous study [19, 21, 40], pH 5.7 precipitation of plant proteins, especially ribulose bisphosphate carboxylase (Rubisco), was used in an attempt to partially purify NVCP. However, utilizing a similar strategy resulted in substantial loss of NaVCP as compared to NVCP. When the pH of the extraction buffer was raised from pH 5.7 to pH 6.6, the concentration of extracted NaVCP was much higher in the soluble fraction (Table 2) and minimal loss (less than 10%) was observed in the insoluble pellet (data not shown). The supernatant was concentrated by filtering through a 300 kDa cut-off membrane to remove some of the plant endogenous proteins followed by 30 kDa cut-off membrane to concentrate the extracted NaVCP.

**Figure 3:**
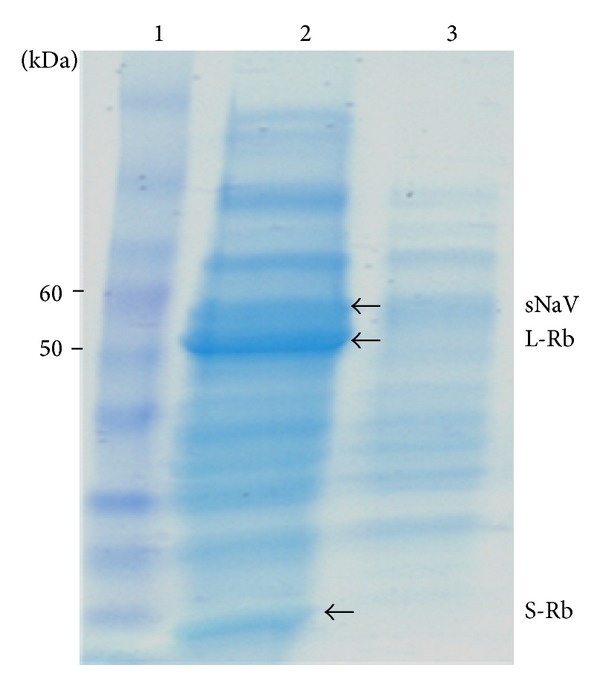
Partial purification of NaVCP expressed in* N. benthamiana* leaf. Coomassie stained gel showing partially purified NaVCP after DEAE chromatography. Lane 1: protein molecular mass markers; lane 2: concentrated plant extract before DEAE chromatography; lane 3: DEAE chromatography flow-through containing NaVCP. sNaV: Narita virus capsid protein; L-Rb: large subunit of Rubisco; S-Rb: small subunit of Rubisco.

The final concentrated extract was further fractionated using anion exchange chromatography to yield partially purified NaVCP extract. The majority of the NaVCP was contained in the flow-through fraction (Figure 3, lane 3), while the two major endogenous plant proteins, large and small subunit of Rubisco, which comprise ~50% of the leaf protein, were lacking in the flow-through fraction. Rubisco subunits were finally eluted in the high salt buffer. The flow-through fractions were pooled, concentrated further, and used for immunization studies. The DEAE purified NaVCP used for animal studies was ~5% of total soluble protein (TSP) as measured by ELISA.

### 3.4. Intranasal Vaccination with NaVCP Induces Serum and Mucosal VLP-Specific Antibody Responses

**Figure 4:**
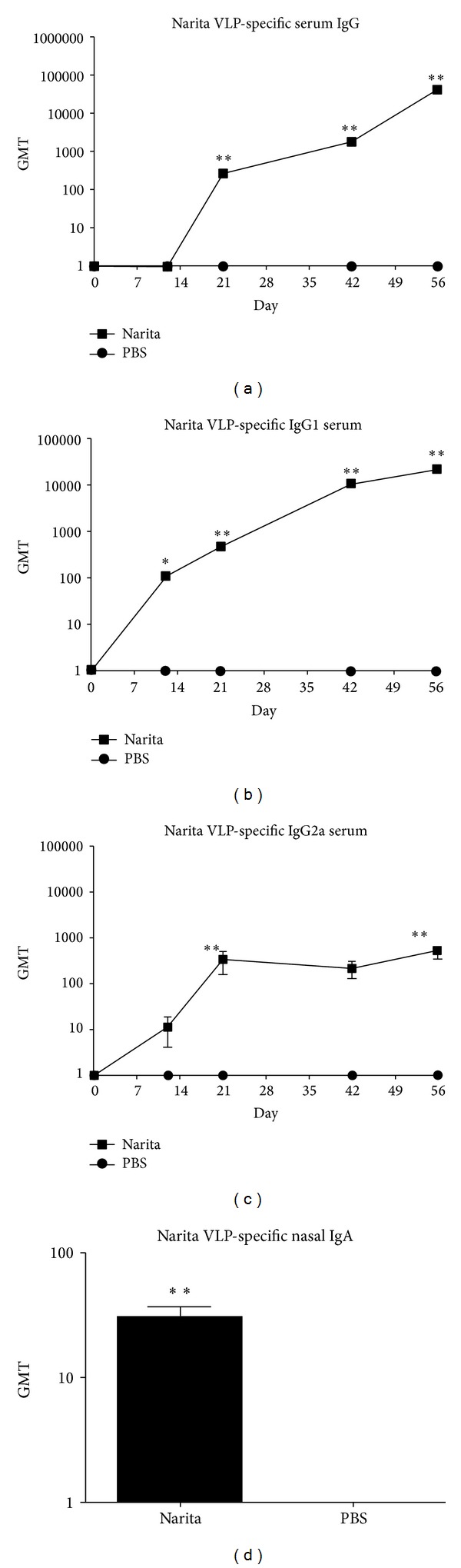
Intranasal immunization with NaVLP induced significant serum and mucosal VLP-specific antibody production in mice. Female BALB/c mice (*n* = 10 per group) received intranasal delivery of plant-derived NaVCP [25 *μ*g] on day 0 and day 21. Time course analysis of VLP-specific serum IgG and IgG isotypes ((a)–(c)) and nasal IgA (d) responses in mice following intranasal delivery of plant-derived NaVLP. Serum IgG, IgG isotypes, and nasal IgA were measured by ELISA. The *y*-axis shows the geometric mean titers (GMTs) and the error bars show the standard error of the mean. A* P* value of <0.05 was considered significant. *< 0.01 and **< 0.001.

To evaluate the immunogenicity of the plant-derived NaVCP, female mice were intranasally vaccinated with NaVLP (25 *μ*g) or mock-vaccinated (PBS treated) on days 0 and 21. Serum was collected on days 0, 12, 21, 42, and 56 after vaccination and evaluated for VLP-specific antibody production. Nasal washes were collected at the end of the study (day 56) following euthanasia. By day 21, a significant amount of Narita VLP-specific antibodies was produced in the antigen-containing group relative to the mock PBS controls (Figure 4(a)). The amount of VLP-specific IgG and IgG isotype antibody in the serum continued to rise over the period of the study through day 56 (Figures 4(a)–4(c)). Consistent with studies using NV VLP alone (without adjuvant), IgG1 levels were much higher relative to the IgG2a isotype indicating a predominately Th2 response [38, 41]. A significant amount of VLP-specific nasal IgA production was observed in the NaVCP vaccinated mice relative to the mock-vaccinated controls (Figure 4(d)), indicating a robust local response. Thus, NaVLP delivered intranasally resulted in significant levels of VLP-specific antibody at serum and mucosal sites (Figure 4).

## 4. Discussion

VLPs have been used as vaccines as a safer alternative to attenuated live or inactivated killed viruses. VLP-based vaccines have been successful in the clinic; for example, there are two commercially available HPV VLP-based vaccines [42]. Recombinant Norwalk (rNV) VLPs are being investigated as vaccine candidates in the clinic as the VLPs are morphologically and antigenically similar to Norwalk virus as demonstrated by electron microscopy and ELISA [17, 20, 43, 44]. Initial studies using insect-derived rNV VLP expressed via baculovirus expression system were immunogenic in mice [17, 20, 45]. Using adjuvants and formulations can significantly enhance the mucosal immunogenicity of plant-based VLP vaccines as recently shown [38, 41, 46].

For the last decade, plant-based vaccines have gained steady acceptance in the scientific community. Plant-based expression is a cost-effective and convenient system for antigens to be used in mucosal delivery. Plants represent relatively safe platforms to express vaccines since they are free of mammalian pathogens that affect other production systems such as transgenic animals and cell lines [29, 30]. Norwalk VLPs have been previously shown to accumulate in transgenic potato [18], tobacco [18, 21], and tomato [40, 47]. Very high expression (0.8 mg/g leaf) of NV VLPs was attained in transient assay using viral vectors TMV-based expression system in* N. benthamiana *[21]. In this study, feasibility of high-level expression of norovirus Narita 104 was explored for use as subunit vaccine. Narita 104 virus is a GII.4 strain of norovirus similar to those responsible for recent outbreaks [48].

NaVCP was expressed using a replicating plant virus based strategy to yield high levels of protein expression [24]. In this strategy, NaVCP was inserted among viral replicating elements, amplified episomally, and subsequently translated in the cytosol of plant cell. Expression in* N. benthamiana* required partial purification of the protein prior to using it in immunization studies. A high level of expression of foreign protein (0.5–5 mg recombinant protein per g of leaf biomass) has been obtained with the TMV based system with no signs of necrosis on the infiltrated plants [49]. NVCP, which is similar to NaVCP, was expressed at 0.8 mg/g leaf in* N. benthamiana* at 12 DPI and necrosis was not observed even after 22 DPI [21]. However, expression of NaVCP was limited due to rapid emergence of a hypersensitive response-like symptom followed by cell death by 5 DPI. In spite of this shorter infection time, expression of NaVCP was reasonably high, attaining 0.3 mg/g leaf. A similar response was observed when expression of NaVCP was driven by a geminiviral vector system [39] in* N. benthamiana *(Mathew, LG., and Mason HS., unpublished data). In spite of the cell death response, expression observed with the TMV system was approximately 55-fold higher than the transient expression in* N. benthamiana* leaf using a nonreplicating vector system (Mathew, LG., and Mason, HS. unpublished data).

The rapid induction of a leaf cell death response by NaVCP is interesting, since the related NVCP showed no such response [21]. The capsid proteins of Norwalk and Narita 104 viruses are substantially divergent, with amino acid sequences 45% identical and 60% similar over the entire length. The capsid proteins have similar protein structures comprising shell (S) and protruding (P) domains [50]. The S domains are more highly conserved, and the P1 subdomains are moderately well conserved. However, the most surface-exposed P2 subdomains are the most variable, and NaVCP has a 10-aa insertion “QTGQNTKFTP.” We performed P2 subdomain swapping experiments; however, when the chimeric capsid proteins were expressed in* N. benthamiana, *the results were inconclusive and failed to show convincing evidence of the direct involvement of P2 in the induction of cell death by NaVCP (Mathew and Mason, unpublished data). Nonetheless, it seems likely that the sequence divergence is related to the great difference in induction of the cell death response and could be mediated by host cell defense responses that recognize a particular pattern in NaVCP.

The evidence that plant-derived NaVCP assembles into NaVLP strengthens the finding that plant-derived NaVCP can be used as a vaccine candidate. The observation that plant- and insect-derived NaVLP showed different sucrose gradient sedimentation profiles may be explained in several ways. The electron micrograph of insect-derived NaVLP showed that most particles appeared to be either partially assembled, characterized by a deeper electron dense stain, or small 20 nm VLP (Figure 2(b) right). The plant-derived NaVLP appeared to be mostly fully assembled with rare occurrence of 20 nm VLPs (Figure 2(b) left). The partially assembled and smaller 20 nm VLPs were likely slower sedimenting than the plant-derived VLPs. Another possibility is that plant-derived VLP formed aggregates, perhaps by interaction with endogenous cellular material and, therefore, sedimented faster. Further studies need to be conducted in order to understand the difference in the sedimentation pattern from insect-derived and plant-derived NaVLP.

In spite of the relatively shorter infection period due to induction of hypersensitive response, useful yields of NaVCP were obtained. The crude extract had a significantly large amount of endogenous plant protein, including Rubisco, which constitutes ~50% of the total leaf protein [51]. A strategy using pH 5.7 extraction buffer [21], resulted in substantial loss (almost half) of the NaVCP protein in the supernatant (Table 2). The size exclusion concentration aided in the removal of plant endogenous protein and some partially assembled particles. However, the final concentrate did show a substantial amount of Rubisco, both large and small subunits (Figure 3, lane 2). Most of the Rubisco was removed in the final anion exchange chromatography by maintaining the buffer at acidic pH (Figure 3, lane 3).

The partially purified NaVLPs were intranasally delivered to mice to determine the level of immunogenicity both systemically and at the local mucosal immunization site. In the absence of adjuvant, nasal immunization resulted in the significant induction of VLP-specific antibodies in serum and nasal secretions. Therefore, this GII.4-specific vaccine candidate was immunogenic, thus validating the use of plant-derived NaV as a norovirus vaccine or component for a multivalent formulation. Further, we have seen no difference in antibody responses produced by insect cell-derived and plant-derived norovirus VLP prepared similarly and delivered at comparable doses (data not shown). Another indication of the biosimilarity of plant and insect cell-derived VLP is that mice immunized with tomato-derived Norwalk VLP were strongly boosted using insect cell-derived VLP [39].

Although the VLP sample was relatively impure (Figure 3), the robust immune responses in vaccinated mice suggest that contaminating leaf proteins did not impair the immunogenicity of VLP. Additional studies will need to be conducted to optimize the purification, formulation, delivery route, and immunogenicity of this VLP-based vaccine candidate. There is no small animal model or in vitro propagation assay for human norovirus; therefore, it would be difficult to determine if the antibody levels generated in this study would correlate to protection from challenge. For the purpose of this study we aimed to determine the immunogenicity of the GII-specific norovirus VLP vaccine candidate. Future studies will optimize this formulation and test for blockade of VLP binding to histoblood group antigens specific for this genogroup of norovirus.

In conclusion, our plant-based expression technology provided a convenient platform for expedient and cost-effective production of immunogenic recombinant NaVLPs for vaccine studies. Future preclinical studies that include plant-derived GII-4-specific VLP within a multivalent vaccine will be required to determine if this approach can be exploited for generating a protective norovirus vaccine formulation.

## Figures and Tables

**Table 1:** NaVCP accumulation in *N. benthamiana *leaf at different times after infiltration with *Agrobacteria*.

DPI	*μ*g/g fresh wt.	% NaVCP/TSP
3	198 ± 11	1.30 ± 0.10
4	285 ± 3	1.89 ± 0.25
5	Necrosis observed	

DPI: days postinfiltration.

**Table 2:** Extraction efficiency of NaVCP using different pH for buffer.

pH	*μ*g/g fresh wt.	% NaVCP/TSP
5.7	53 ± 18	0.7 ± 0.15
6.6	133 ± 22	1.4 ± 0.16
